# Ring Cleavage Reactions of Methyl α-D-Allopyranoside Derivatives with Phenylboron Dichloride and Triethylsilane

**DOI:** 10.3390/molecules161210303

**Published:** 2011-12-13

**Authors:** Masaru Kojima, Yutaka Nakamura, Yuusuke Ito, Seiji Takeuchi

**Affiliations:** Niigata University of Pharmacy and Applied Life Sciences, 265-1 Higashijima, Akiha-ku, Niigata 956-8603, Japan

**Keywords:** methyl α-D-allopyranoside, endocyclic cleavage, phenylboron dichloride, triethylsilane

## Abstract

In the course of our studies on the regioselective carbon-oxygen bond cleavage of the benzylidene acetal group of hexopyranosides with a reducing agent, we found that a combination of a Lewis acid and a reducing agent triggered a ring-opening reaction of the pyranose ring of methyl α-D-allopyranosides. The formation of an acyclic boronate ester by the attachment of a hydride ion at C-1 indicated that the unexpected endocyclic cleavage of the bond between the anomeric carbon atom and the pyranose ring oxygen atom proceeded via an oxacarbenium ion intermediate produced by the chelation between O5/O6 of the pyranoside and the Lewis acid, followed by nucleophile substitution with a hydride ion at C1.

## 1. Introduction

Lewis-acid-induced regioselective carbon-oxygen bond cleavage of the benzylidene acetal group of hexopyranosides with a reducing agent is an important reaction in carbohydrate chemistry for the syntheses of complex oligosaccharides and glycoconjugates. Until now, various reagent systems [1,2,3,4,5,6,7,8,9,10,11,12] and investigations of the detailed mechanistic pathway [13,14] have been reported for the regioselective reduction of 4,6-*O*-benzylidene acetal groups.

**Scheme 1:**
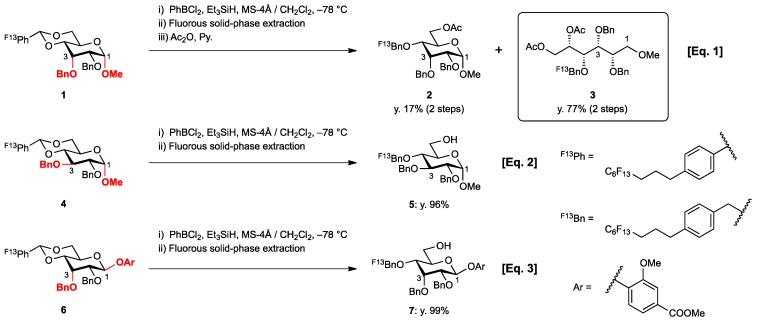
Reductive cleavage of fluorous benzylidene acetal group using PhBCl_2_/Et_3_SiH.

Recently, we reported the synthesis of a new fluorous benzylidene acetal group for the protection of 1,3-diol compounds [15]. Efficient and expeditious syntheses of natural products [16], oligosaccharides [15], and modified monosaccharides have been accomplished by utilizing regioselective ring-opening reduction of fluorous benzylidene acetal groups and solid-phase extraction with a fluorous reverse-phase silica gel column. In the course of our studies on the expeditious synthesis of these products using fluorous benzylidene acetal groups, we isolated an interesting side product, the acyclic compound **3**, during the regioselective ring-opening reduction of methyl 2,3-di-*O*-benzyl-4,6-*O*-^F^benzylidene-α-D-allopyranoside **1** with PhBCl_2_/Et_3_SiH (Scheme 1, Eq. 1). This unexpected side reaction is caused by the reductive cleavage of the fluorous benzylidene acetal group and subsequent endocyclic cleavage of the pyranosides. When methyl 2,3-di-*O*-benzyl-4,6-*O*-^F^benzylidene-α-D-glucopyranoside **4** and phenyl 2,3-di-*O*-benzyl-4,6-*O*-^F^benzylidene β-D-allopyranoside **6** were reacted under the same reaction conditions, this unexpected side reaction was not observed (Scheme 1, Eqs. 2,3).

Only a few reports have been published so far on the anomerization [17,18,19,20,21,22,23,24] and attachment of nucleophiles at C1 [25,26,27,28,29,30,31,32] via the endocyclic cleavage of glycosides. To the best of our knowledge, the side reaction described here is the first example of the endocyclic cleavage of methyl α-D-allopyranoside derivatives with PhBCl_2_/Et_3_SiH. Here, we provide detailed results of the ring cleavage reaction of hexopyranosides bearing axial substituents at C1 and C3.

## 2. Results and Discussion

Initially, methyl 2,3-di-*O*-benzyl-4,6-*O*-benzylidene-α-D-allopyranoside **8** was reacted with PhBCl_2_ (5.0 equiv.) and Et_3_SiH (4.5 equiv.) in CH_2_Cl_2_ at −78 °C. The purification of the crude product by silica gel column chromatography unexpectedly gave an acyclic derivative bearing a boronate ester as the main product [33]. Thus, to remove the phenylboronate group from the acyclic alditol derivative, an octadecyl silica gel (ODS) column was used instead of a fluorous reverse-phase silica gel column. The crude product was loaded onto the ODS column, after which the column was eluted successively with 40% aq. MeOH and then with MeOH. The methanol fraction subsequently was evaporated, and the residue was treated with Ac_2_O and pyridine to give acyclic derivative **19** in 78% yield. In the case of methyl β-D-allopyranoside **11**, the acyclic derivative **19** and 4-*O*-benzyl derivative **23** were obtained in 17% and 46% yields, respectively. However, the endocyclic cleavage of methyl α-D-glucopyranoside **12**and methyl α-D-galactopyranoside **13** was not observed. These results suggest that the hexopyranoside bearing axial substituents at C1 and C3 preferentially undergo endocyclic cleavage. To test the generality of this new finding, we examined the ring opening of various hexopyranosides bearing axial substituents at C1 and C3 under the same reaction conditions. The results are summarized in Table 1. When the reactions were carried out using methyl α-D-allopyranoside derivatives **9** and **10** bearing methoxymethyl ethers and benzoyl esters at C2 and C3, the number of spots observed by thin-layer chromatography (TLC) was so large that the spots could not be identified. In the cases of methyl α-D-gulopyranoside **14**, allyl α-D-allopyranoside **15**, and methyl α-D-*ribo*-hexopyranoside **16**, the reactions proceeded smoothly to give the desired acyclic compounds **26**, **27**, and **28** in high yields. Additionally, the reaction involving hexopyranosides **17** and **18** bearing an axial substituent at C2 gave the acyclic compound **29** and the 4-*O*-benzylated compound **30** in 27% and 83% yields, respectively.

**Table 1:** Synthesis of acyclic derivatives from alkyl 4,6-*O*-benzylidene-α-D-hexopyranosides. 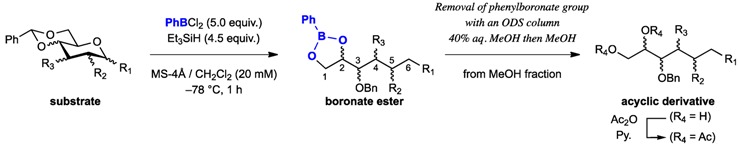

Entry	Substrate	Product (isolated yield)	
		Acyclic derivative	4- *O*-benzylated derivative
	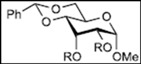	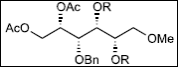	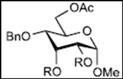
1	**8**: R = Bn	**19** (R = Bn): y. 78% ^a^	**20** (R = Bn): y. 7% ^a^
2	**9**: R = MOM	**21** (R = MOM): y. - ^b^	
3	**10**: R = Bz	**22** (R = Bz): y. - ^b^	
4	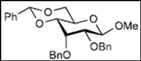 **11**	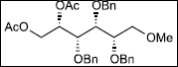 **19**: y. 17%	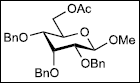 **23**: y. 46%
5	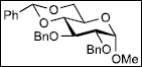 **12**	-	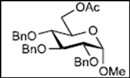 **24**: y. 78%
6	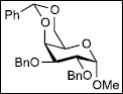 **13**	-	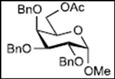 **25**: y. 80%
7	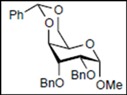 **14**	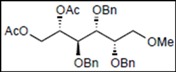 **26**: y. 71%	-
8	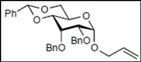 **15**	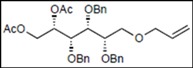 **27**: y. 77%	-
9	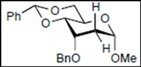 **16**	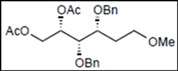 **28**: y. 86%	-
	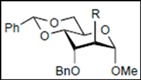	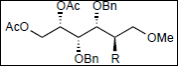	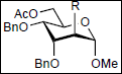
10	**17**: R = OBn	**29** (R = OBn): y. 27% ^c^	
11	**18**: R = N_3_	(R = N_3_): -	**30** (R = N_3_): y. 83%

^a^ When 3.4 equiv. of PhBCl_2_ and 3.0 equiv. of Et_3_SiH were used, acyclic compound **19** and 4-*O*-benzylated compound **20** were obtained in 59% and 19% yields, respectively; ^b^ Many spots were observed by TLC; ^c^ An inseparable mixture was obtained as a main product.

We expected the hydroxyl group at C6 of the hexopyranosides to play an important role in cleavage of the bond between the anomeric carbon C1 and the pyranose ring oxygen atom O5 during endocyclic cleavage of hexopyranosides bearing axial substituents at C1 and C3 with PhBCl_2_/Et_3_SiH because a 1,2-boronate ester derivative was isolated as an intermediate. Therefore, methyl 2,3,4,6-tetra-*O*-benzyl-α-D-allopyranoside **31** and methyl 2,3,4-tri-*O*-benzyl-α-D-allopyranoside **32** were reacted with PhBCl_2_ and Et_3_SiH. As shown in Table 2, compound **31** gave methyl 3,4,6-tri-*O*-benzyl-α-D-allopyranoside **33** and the starting material **31** in 28% and 52% yields, respectively. Although the reaction of 6-hydroxy-derivative **32** at −78 °C gave the desired acyclic derivative **34** with only a 10% yield, the yield reached 92% when the reaction was carried out at −19 °C.

**Scheme 2:**
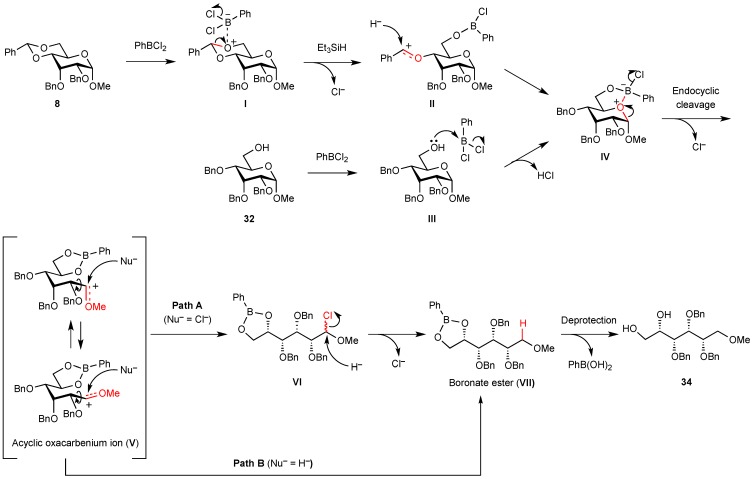
Proposed reaction mechanism.

On the basis of these experimental data, the pathway for PhBCl_2_-induced endocyclic cleavage of hexopyranosides with 1,3-diaxial substituents is speculated to be that shown in Scheme 2. The endocyclic cleavage is initiated by bond formation between the boron atom and oxygen atom O6 followed by chelation of the boron atom at ring oxygen atom O5. This interaction promotes cleavage of the endocyclic C1-O5 bond and formation of acyclic oxacarbenium ion **V**. Before or after rotation around the C1-C2 bond, the addition of chloride ion from PhBCl_2_ to cation **V** followed by nucleophilic substitution with hydride ion (Path A) or direct addition of hydride ion to cation **V** (Path B) gives boronate ester **VII**. The major factor in the endocyclic cleavage of methyl α-D-allopyranoside **8** is due to steric strain of pyranosidic ring caused by steric repulsions between the substituents at C1 and C-3. Hexopyranosides **12** and **13** in which the pyranosidic rings are stabilized by the equatorial substituent at C-3 do not produce the corresponding acyclic derivatives. In the case of the reaction of hexopyranoside **11**, the equatorial methoxy group at C-1 sterically hinders bond formation between the boron atom and O5/O6 to give alditol derivative **19** in low yield. Since the ^4^C_1_ conformation of altropyranoside **17** or **18** bearing axial substituents at C1, C2, and C3 is rapidly converted into the more stable ^1^C_4_ conformation in which all the substituents are equatorial after the benzylidene acetal group is cleaved, the altropyranosides give alditol derivative **29** in low yield and 4-*O*-benzylated compound **30** in high yield. The endocyclic cleavage of 6-hydroxy-derivative **32** at −78 °C results in the lower yield because the formation of **IV** is inhibited at the lower temperature, although the reaction from **III** to **IV** proceeds smoothly at the higher temperature.

However, the above-mentioned mechanism is highly speculative because of the lack of enough experimental data for supporting it. Therefore, we are now making efforts to get essential data for clarifying the mechanism by several experiments. We will report the results in the near future.

**Table 2:** Synthesis of acyclic derivatives from methyl 2,3,4,6-tetra-*O*-benzyl-α-D-allopyranoside and 2,3,4-tri-*O*-benzyl-α-D-allopyranoside. 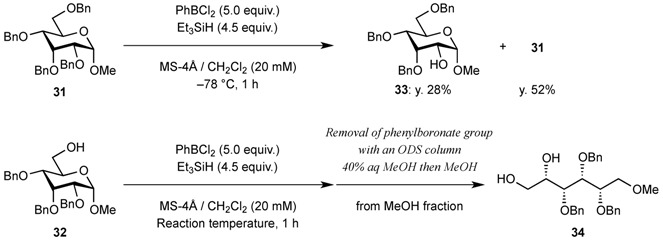

Entry	Reaction temperature (°C)	Yield of acyclic compound 34 (%)	Yield of recovered starting material 32 (%)
1	−78	10	80
2	−60	53	41
3	−50	70	24
4	−40	80	18
5	−19	92	-

## 3. Experimental

### 3.1. General

^1^H- and ^13^C-NMR spectra were measured using a Bruker Avance DPX-250 spectrometer. *J* values were recorded in Hertz, and the abbreviations used were s (singlet), d (doublet), t (triplet), m (multiplet), and br (broad). Chemical shifts are expressed in *δ* values relative to the internal standard TMS. Octadecyl silica gel column chromatography was carried out using COSMOSIL 75C_18_-OPN (75 μm, Nacalai Tesque) column. TLC was carried out on Merck silica gel 60 F254 plates. PhBCl_2_ and Et_3_SiH were obtained from Sigma-Aldrich and Acros Organics, respectively.

### 3.2. General Procedure for Endocyclic Cleavage with PhBCl_2_ and Et_3_SiH

A suspension of methyl 2,3-di-*O*-benzyl-4,6-*O*-benzylidene-α-D-allopyranoside **8** (50 mg, 0.108 mmol) and MS-4Å (250 mg) in dry CH_2_Cl_2_ (5.4 mL) was stirred for 1 h at room temperature under argon. Next, Et_3_SiH (77 μL, 0.486mmol, 4.5 equiv.) was added to the suspension at −78 °C, after which a solution of PhBCl_2_ (70 μL, 0.541 mmol, 5.0 equiv.) in CH_2_Cl_2_ (1 mL) was added over 1 h via a syringe pump. After stirring for 1 h at the same temperature, the reaction mixture was quenched with Et_3_N (0.5 mL) and MeOH (0.5 mL) and then filtered through Celite. The filtrate was subsequently washed with saturated NaHCO_3_ solution (5 mL) and brine (5 mL), dried over Na_2_SO_4_, filtered, and concentrated. The residue was then loaded onto an octadecyl silica gel column, which was eluted successively with 40% aq. MeOH and MeOH. Next, the MeOH fraction was concentrated to give the residue containing the acyclic diol. The residue was then redissolved in pyridine (0.5 mL), after which acetic anhydride (0.5 mL) was added. After stirring for 3 h at room temperature, the reaction mixture was poured into MeOH at 0 °C and stirred for 10 min. The mixture was evaporated and co-evaporated with toluene. Finally, the residue was subjected to preparative thin-layer chromatography(hexane/EtOAc = 3:2 v/v) to give allitol derivative **19** (46.7 mg, 78% yield).

*(2S,3R,4R,5S)-1,2-Bis(acetoxy)-3,4,5-tris(benzyloxy)-6-methoxyhexane* (**19**). Colorless syrup; *R*_f_ = 0.59 (hexane/EtOAc = 3:2 v/v); IR (NaCl, neat): 1745 cm^−1^; ^1^H-NMR (250 MHz, CDCl_3_): δ 7.33−7.25 (15H, m, Ar*H*), 5.48 (1H, ddd, *J*_2,3_ = 4.0 Hz, *J*_2,1_ = 2.7 Hz, *J*_2,1′_ = 7.3 Hz, H-2), 4.73, 4.68 (2H, each d, *J* = 11.3 Hz, PhC*H_2_*), 4.71, 4.58 (2H, each d, *J* = 11.6 Hz, PhC*H_2_*), 4.60 (2H, s, PhC*H_2_*), 4.43 (1H, dd, *J*_1,2_ = 2.7 Hz, *J*_1,1′_ = 12.2 Hz, H-1), 4.24 (1H, dd, *J*_1′,2_ = 7.3 Hz, *J*_1,1′_ = 12.2 Hz, H-1′), 3.93 (1H, dd t-like, *J*_3,2_ = 4.0 Hz, *J*_3,4_ = 4.4 Hz, H-3), 3.84 (2H, m, H-4, 5), 3.61 (1H, dd, *J*_6,5_ = 3.0 Hz, *J*_6,6′_ = 10.4 Hz, H-6), 3.54 (1H, dd, *J*_6′,5_ = 4.7 Hz, *J*_6′,6_ = 10.4 Hz, H-6′), 3.30 (3H, s, OC*H_3_*), 1.99, 1.97 (6H, each s, C*H_3_* × 2); ^13^C-NMR (63 MHz, CDCl_3_): δ 170.7, 169.8, 138.4, 137.9, 137.7, 128.3, 128.24, 128.22, 128.17, 128.08, 128.03, 127.97, 127.7, 127.63, 127.57, 127.4, 78.4, 78.2, 78.1, 73.8, 72.8, 72.3, 71.85, 71.79, 63.3, 58.9, 21.0, 20.7.

*(2S,3S,4R,5S)-1,2-Bis(acetoxy)-3,4,5-tris(benzyloxy)-6-methoxyhexane* (**26**). Colorless syrup; *R*_f_ = 0.63 (hexane/EtOAc = 3:2 v/v); IR (NaCl, neat): 1744 cm^−1^; ^1^H-NMR (250 MHz, CDCl_3_): δ 7.39−7.20 (15H, m, Ar*H*), 5.38 (1H, ddd, *J*_2,3_ = 5.1 Hz, *J*_2,1_ = 3.6 Hz, *J*_2,1′_ = 7.1 Hz, H-2), 4.77 (2H, each d, *J* = 11.4 Hz, PhC*H_2_*), 4.66, 4.44 (2H, each s, *J* = 11.8 Hz, PhC*H_2_*), 4.64 (2H, s, PhC*H_2_*), 4.30 (1H, dd, *J*_1,2_ = 3.6 Hz, *J*_1,1′_ = 12.0 Hz, H-1), 4.05 (1H, dd, *J*_1′,2_ = 7.1 Hz, *J*_1′,1_ = 12.0 Hz, H-1), 3.92−3.77 (3H, m, H-3, 4, 5), 3.74 (1H, dd, *J*_6,5_ = 3.6 Hz, *J*_6,6′_ = 10.1 Hz, H-6), 3.59 (1H, dd, *J*_6′,5_ = 4.0 Hz, *J*_6′,6_ = 10.1 Hz, H-1′), 3.35 (3H, s, OC*H_3_*), 2.01, 1.97 (6H, each s, C*H_3_* × 2); ^13^C-NMR (63 MHz, CDCl_3_): δ 170.5, 170.2, 138.4, 138.3, 138.0, 128.31, 128.29, 128.0, 127.9, 127.7, 127.60, 127.58, 127.52, 78.5, 78.3, 77.0 (overlapped with CDCl_3_), 74.5, 73.9, 72.0, 71.3, 71.2, 63.0, 58.9, 20.9, 20.7.

*(2S,3R,4R,5S)-6-(Allyloxy)-1,2-bis(acetoxy)-3,4,5-tris(benzyloxy)hexane* (**27**). Colorless syrup; *R*_f_ = 0.50 (hexane/EtOAc = 3:2 v/v); IR (NaCl, neat): 1744 cm^−1^; ^1^H NMR (250 MHz, CDCl_3_): δ 7.34−7.24 (15H, m, Ar*H*), 5.87 (1H, ddt, *J* = 5.5 Hz, *J* = 10.4 Hz, *J* = 17.2 Hz, CH_2_C*H*=CH_2_), 5.49 (1H, ddd, *J*_2,3_ = 3.8 Hz, *J*_2,1_ = 2.7 Hz, *J*_2,1′_ = 7.3 Hz, H-2), 5.23 (1H, dq, *J* = 1.6 Hz, *J* = 17.2 Hz, CH_2_CH=C*H_2_*), 5.14 (1H, dq, *J* = 1.3 Hz, *J* = 10.4 Hz, CH_2_CH=C*H_2_*), 4.72, 4.60 (2H, each d, *J* = 11.7 Hz, PhC*H_2_*), 4.70, 4.60 (4H, each s, PhC*H_2_* × 2), 4.42 (1H, dd, *J*_1,2_ = 2.7 Hz, *J*_1,1′_ = 12.2 Hz, H-1), 4.24 (1H, dd, *J*_1′,2_ = 7.3 Hz, *J*_1′,1_ = 12.2 Hz, H-1′), 3.95−3.82 (5H, m, H-3, 4, 5, C*H_2_*CH=CH_2_), 3.68 (1H, dd, *J*_6,5_ = 3.0 Hz, *J*_6,6′_ = 10.4 Hz, H-6), 3.59 (1H, dd, *J*_6′,5_ = 5.2 Hz, *J*_6′,6_ = 10.4 Hz, H-6), 1.99, 1.97 (6H, each s, C*H_3_* × 2); ^13^C-NMR (63 MHz, CDCl_3_): δ 170.7, 169.8, 138.6, 138.0, 137.8, 134.8, 128.29, 128.26, 128.21, 128.12, 128.05, 127.8, 127.7, 127.6, 127.4, 116.7, 78.6, 78.4, 78.3, 73.8, 72.9, 72.5, 72.2, 71.9, 69.7, 63.3, 21.0, 20.8.

*(2S,3S,4R)-1,2-Bis(acetoxy)-3,4-bis(benzyloxy)-6-methoxyhexane* (**28**). Colorless syrup; *R*_f_ = 0.55 (hexane/EtOAc = 1:1 v/v); IR (NaCl, neat): 1745 cm^−1^; ^1^H-NMR (250 MHz, CDCl_3_): δ 7.38−7.25 (10H, m, Ar*H*), 5.28 (1H, ddd, *J*_2,3_ = 4.8 Hz, *J*_2,1_ = 2.6 Hz, *J*_2,1′_ = 6.9 Hz, H-2), 4.72, 4.55 (2H, each d, *J* = 11.5 Hz, PhC*H_2_*), 4.68, 4.63 (2H, each d, *J* = 10.5 Hz, PhC*H_2_*), 4.48 (1H, dd, *J*_1,2_ = 2.6 Hz, *J*_1,1′_ = 12.2 Hz, H-1), 4.25 (1H, dd, *J*_1′,2_ = 6.9 Hz, *J*_1′,1_ = 12.2 Hz, H-1′), 3.80−3.73 (2H, m, H-3, 4), 3.54−3.35 (2H, m, H-6, 6′), 3.26 (3H, s, OC*H_3_*), 2.04, 2.01 (6H, each s, C*H_3_* × 2), 1.92−1.84 (2H, m, H-5); ^13^C-NMR (63 MHz, CDCl_3_): δ 170.6, 169.9, 138.2, 137.9, 128.3, 128.0, 127.7, 127.6, 79.3, 75.9, 73.3, 72.5, 71.4, 68.8, 63.2, 58.4, 30.6, 20.9, 20.7.

*(2S,3R,4R,5R)-1,2-Bis(acetoxy)-3,4,5-tris(benzyloxy)-6-methoxyhexane* (**29**). Colorless syrup; *R*_f_ = 0.46 (hexane/EtOAc = 3:2 v/v); IR (NaCl, neat): 1744 cm^−1^; ^1^H-NMR (250 MHz, CDCl_3_): δ 7.67−7.20 (15H, m, Ar*H*), 5.40 (1H, ddd, *J*_2,3_ = 3.4 Hz, *J*_2,1_ = 2.8 Hz, *J*_2,1′_ = 7.3 Hz, H-2), 4.79, 4.70 (2H, each d, *J* = 11.3 Hz, PhC*H_2_*), 4.66, 4.58 (2H, each d, *J* = 11.7 Hz, PhC*H_2_*), 4.62, 4.51 (2H, each d, *J* = 11.6 Hz, PhC*H_2_*), 4.54 (1H, dd, *J*_1,2_ = 2.8 Hz, *J*_1,1′_ = 12.2 Hz, H-1), 4.27 (1H, dd, *J*_1′,2_ = 7.3 Hz, *J*_1′,1_ = 12.2 Hz, H-1′), 3.93−3.85 (2H, m, H-3, 4), 3.79 (1H, ddd q-like, *J*_5,4_ = 4.8 Hz, *J*_5,6_ = 4.8 Hz, *J*_5,6′_ = 4.8 Hz, H-5), 3.57 (1H, dd, *J*_6,5_ = 4.7 Hz, *J*_6,6′_ = 10.2 Hz, H-6), 3.51 (1H, dd, *J*_6′,5_ = 4.8 Hz, *J*_6′,6_ = 10.2 Hz, H-6′), 3.30 (3H, s, OC*H_3_*), 1.98 (6H, s, C*H_3_* × 2); ^13^C-NMR (63 MHz, CDCl_3_): δ 170.7, 169.9, 138.6, 138.3, 137.9, 128.31, 128.28, 128.26, 128.1, 127.9, 127.8, 127.7, 127.6, 127.5, 78.8, 78.2, 74.6, 73.0, 72.7, 72.0, 71.9, 63.3, 59.1, 21.0, 20.8.

*(2S,3R,4R,5S)-1,2-Dihydroxy-3,4,5-tris(benzyloxy)-6-methoxyhexane* (**34**). Colorless syrup; *R*_f_ = 0.24 (hexane/EtOAc = 3:2 v/v); IR (NaCl, neat): 3444 cm^−1^; ^1^H-NMR (250 MHz, CDCl_3_): δ 7.38−7.25 (15H, m, Ar*H*), 4.73, 4.61 (2H, each d, *J* = 11.6Hz, PhC*H_2_*), 4.71 (2H, s, PhC*H_2_*), 4.68, 4.55 (2H, each d, *J* = 11.4 Hz, PhC*H_2_*), 3.97 (1H, 1H, dd, *J*_4,3_ = 3.5 Hz, *J*_4,5_ = 6.1 Hz, H-4), 3.90 (1H, ddd, *J*_5,4_ = 6.1 Hz, *J*_5,6_ = 3.5 Hz, *J*_5,6’_ = 4.6 Hz, H-5), 3.93−3.84 (1H, m, H-2, overlapped with H-5), 3.76 (1H, dd, *J*_3,4_ = 3.5 Hz, *J*_3,2_ = 7.0 Hz, H-3), 3.71−3.60 (2H, m, H-1, 1′, overlapped with H-6, 6′), 3.66 (1H, dd, *J*_6,5_ = 3.5 Hz, *J*_6,6′_ = 10.4 Hz, H-6), 3.59 (1H, dd, *J*_6′,5_ = 4.6 Hz, *J*_6′,6_ = 10.4 Hz, H-6′), 3.35 (3H, s, OC*H_3_*), 3.22 (1H, br d, *J* = 3.7 Hz, O*H*), 2.17 (1H, br s, O*H*); ^13^C-NMR (63 MHz, CDCl_3_): δ 138.02, 137.95, 137.90, 128.43, 128.41, 128.08, 128.06, 128.01, 127.9, 127.8, 79.4, 79.3, 78.1, 73.9, 73.2, 72.7, 71.81, 71.78, 63.9, 59.2.

## 4. Conclusions

The reaction of alkyl 4,6-*O*-benzylidene-α-D-allopyranoside, 4,6-*O*-benzylidene-α-D-gulopyranoside, and 4,6-*O*-benzylidene-α-D-altropyranoside derivatives carrying 1,3-diaxial substituents with PhBCl_2_/Et_3_SiH gave 4-*O*-benzyl ethers and alditol derivatives formed by C1/O5 bond cleavage. Because an acyclic boronate ester was isolated, the unexpected endocyclic cleavage is considered to proceed via an oxacarbenium ion intermediate produced by the chelation between O5/O6 of the pyranoside and PhBCl_2_ followed by nucleophilic substitution with a hydride ion at C1. The oxacarbenium ion could be employed as a valuable and versatile intermediate for stereoselective carbon-carbon, carbon-nitrogen, carbon-sulfur, and carbon-oxygen bond formations with a variety of nucleophiles. Further reactivity studies of this endocyclic cleavage are underway in our laboratory. The results of these studies will be reported in the near future.
